# Identification of Immunogenic *Salmonella enterica* Serotype Typhi Antigens Expressed in Chronic Biliary Carriers of *S.* Typhi in Kathmandu, Nepal

**DOI:** 10.1371/journal.pntd.0002335

**Published:** 2013-08-01

**Authors:** Richelle C. Charles, Tania Sultana, Mohammad Murshid Alam, Yanan Yu, Ying Wu-Freeman, Meagan Kelly Bufano, Sean M. Rollins, Lillian Tsai, Jason B. Harris, Regina C. LaRocque, Daniel T. Leung, W. Abdullah Brooks, Tran Vu Thieu Nga, Sabina Dongol, Buddha Basnyat, Stephen B. Calderwood, Jeremy Farrar, Farhana Khanam, John S. Gunn, Firdausi Qadri, Stephen Baker, Edward T. Ryan

**Affiliations:** 1 Division of Infectious Diseases, Massachusetts General Hospital, Boston, Massachusetts, United States of America; 2 Department of Medicine, Harvard Medical School, Boston, Massachusetts, United States of America; 3 International Centre for Diarrhoeal Disease Research, Bangladesh (ICDDR,B), Dhaka, Bangladesh; 4 Department of Biology and Chemistry, Fitchburg State University, Fitchburg, Massachusetts, United States of America; 5 Hospital for Tropical Diseases, Wellcome Trust Major Overseas Programme, Oxford University Clinical Research Unit, Ho Chi Minh City, Vietnam; 6 Oxford University Clinical Research Unit, Patan Academy of Health Sciences, Kathmandu, Nepal; 7 Department of Microbiology and Immunobiology, Harvard Medical School, Boston, Massachusetts, United States of America; 8 Centre for Tropical Medicine and Nuffield Department of Clinical Medicine, Oxford University, Oxford, United Kingdom; 9 Center for Microbial Interface Biology, The Ohio State University, Columbus, Ohio, United States of America; 10 The London School of Hygiene and Tropical Medicine, London, United Kingdom; 11 Department of Immunology and Infectious Diseases, Harvard School of Public Health, Boston, Massachusetts, United States of America; University of California San Diego School of Medicine, United States of America

## Abstract

**Background:**

*Salmonella enterica* serotype Typhi can colonize and persist in the biliary tract of infected individuals, resulting in a state of asymptomatic chronic carriage. Chronic carriers may act as persistent reservoirs of infection within a community and may introduce infection to susceptible individuals and new communities. Little is known about the interaction between the host and pathogen in the biliary tract of chronic carriers, and there is currently no reliable diagnostic assay to identify asymptomatic *S.* Typhi carriage.

**Methodology/Principal Findings:**

To study host-pathogen interactions in the biliary tract during *S.* Typhi carriage, we applied an immunoscreening technique called *in vivo*-induced antigen technology (IVIAT), to identify potential biomarkers unique to carriers. IVIAT identifies humorally immunogenic bacterial antigens expressed uniquely in the *in vivo* environment, and we hypothesized that *S.* Typhi surviving in the biliary tract of humans may express a distinct antigenic profile. Thirteen *S.* Typhi antigens that were immunoreactive in carriers, but not in healthy individuals from a typhoid endemic area, were identified. The identified antigens included a number of putative membrane proteins, lipoproteins, and hemolysin-related proteins. YncE (STY1479), an uncharacterized protein with an ATP-binding motif, gave prominent responses in our screen. The response to YncE in patients whose biliary tract contained *S.* Typhi was compared to responses in patients whose biliary tract did not contain *S.* Typhi, patients with acute typhoid fever, and healthy controls residing in a typhoid endemic area. Seven of 10 (70%) chronic carriers, 0 of 8 bile culture-negative controls (0%), 0 of 8 healthy Bangladeshis (0%), and 1 of 8 (12.5%) Bangladeshis with acute typhoid fever had detectable anti-YncE IgG in blood. IgA responses were also present.

**Conclusions/Significance:**

Further evaluation of YncE and other antigens identified by IVIAT could lead to the development of improved diagnostic assays to identify asymptomatic *S.* Typhi carriers.

## Author Summary

*Salmonella enterica* serotype Typhi is the cause of typhoid fever and infects over 21 million individuals and causes 200,000 deaths each year. With adequate treatment, most patients recover from their acute stage of illness and clear infection. However, a small percentage of *S.* Typhi infected individuals develop a chronic but asymptomatic infection in the biliary tract that can persist for decades. Since *S.* Typhi is a human-restricted pathogen, chronic carriers may act as reservoirs of infection. Correctly identifying and treating asymptomatic chronic carriers could be critical for ultimate control of typhoid fever. Using an immunoscreening technique called *in vivo*-induced antigen technology (IVIAT), we have identified potential biomarkers unique to *S.* Typhi chronic carriers. Further evaluation of these antigens could lead to the development of improved diagnostic assays to detect asymptomatic *S.* Typhi carriers in typhoid endemic zones, and to an improved understanding of the pathogenesis of *S.* Typhi in the chronic carrier state.

## Introduction

*Salmonella enterica* serovars Typhi (*S.* Typhi) and Paratyphi A (*S.* Paratyphi A) are human-specific pathogens, and the predominant cause of enteric (typhoid) fever globally. Enteric fever affects over 21 million people each year, resulting in 200,000 deaths [1]. Infection with *S.* Typhi and *S.* Paratyphi A usually begins with ingestion of contaminated water or food. The pathogens invade the gastrointestinal mucosa, translocate to the lymphoid follicles where they survive and replicate within macrophages, and then disseminate via the bloodstream to the liver, spleen, intestinal lymph nodes, bone marrow, and gallbladder [2]. With adequate treatment, most patients recover from their acute stage of illness and clear infection. However, a small percentage of *S.* Typhi (and *S.* Paratyphi A) infected individuals develop a chronic, but apparently asymptomatic, infection in the biliary tract that can persist for decades [3]–[6]. The likelihood of this is not known, but it is estimated that chronic carriage can complicate perhaps 1–3% of acute infections [7].

Since *S.* Typhi and *S.* Paratyphi A are human-restricted pathogens, chronic carriers may act as reservoirs of infection within a community. They contribute to the transmission cycle through the intermittent shedding of bacteria in feces (especially in areas of low transmission [8]) and may act as vehicles for introducing *S.* Typhi and *S.* Paratyphi A into previously uninfected communities. Therefore, correctly identifying and treating asymptomatic chronic carriers is critical for the long-term control of enteric fever. Currently, there is no reliable diagnostic assay to identify asymptomatic *S.* Typhi and *S.* Paratyphi A carriage. Bacterial stool culture has been used, yet is challenging due to the expense and logistics of obtaining multiple samples from patients, since shedding is typically low level and intermittent [5]. Measurement of antibody responses to the *S.* Typhi capsular Vi antigen has been previously described as a potential method to detect chronic *S.* Typhi carriers [7]. In laboratory settings, IgG to the Vi antigen has been shown to have a sensitivity of 75% and specificity of >95% and has proven to complement other strategies in outbreak investigations [7], [9]–[11]. However, its role in detecting asymptomatic carriers in a general endemic-zone population is unclear. In Chile, anti-Vi antibody responses had a sensitivity of 75% and specificity of 92%–97% for *S.* Typhi carriage; however, due to a low prevalence rate of carriage in the general population, its positive predictive value was only 8–17% [12]. In Vietnam, a large community-based survey for anti-Vi antibodies demonstrated a 3% positivity rate in the population; however, *S.* Typhi was never detected in the stool of individuals identified by such anti-Vi screening [13].

Understanding the mechanisms involved in development and persistence of the carrier state may facilitate development of improved diagnostic assays and therapeutic approaches for *S.* Typhi carriage. Currently, little is known about host-pathogen interactions in the biliary tract of chronic human carriers. Much of what is known about biliary carriage has been extrapolated from *in vitro* and murine studies with *S.* Typhimurium, which causes an enteric fever-like illness in mice [5]. From these animal studies and a complimentary study in humans, we know that gallstones facilitate *S.* Typhi carriage [5]. In the presence of bile, the bacterium regulates the expression of genes that allow it to colonize and persist in the gallbladder through formation of biofilms that mediate resistance against host defenses [14], [15]. There are likely other niches of persistent infection outside of the gallbladder, including the biliary tree, liver, and mesenteric lymph nodes. This is suggested by the observation that although cholecystectomy increases cure rates, it does not always result in clearance of the pathogen in humans [16]. In a murine model of *Salmonella* chronic infection, *S.* Typhimurium infection in Slc11a1 (Nramp1) wild-type mice demonstrated that the most common site of persistent infection was in hemophagocytic macrophages within mesenteric lymph nodes [2], [17], [18].

To advance our understanding of *Salmonella* pathogenesis of the chronic carrier state, and identify potential biomarkers unique to *S.* Typhi chronic carriers, we applied an immunoscreening technique called *in vivo*-induced antigen technology (IVIAT) [19]–[21]. IVIAT identifies humorally immunogenic bacterial antigens expressed *in vivo* and not in bacteria grown in standard laboratory conditions. We hypothesized that *S.* Typhi surviving in the biliary tract of humans may express a proteomic profile distinct from that expressed in bacteria grown using standard *in vitro* conditions or during acute infection.

## Methods

### Ethics statement

This study was approved by the human studies committees of the involved research institutions: Massachusetts General Hospital, International Centre of Diarrheal Disease Research, Bangladesh (icddr,b), Patan Hospital, The Nepal Health Research Council, and the Oxford Tropical Research Ethics Committee. The study was conducted according to the principles expressed in the Declaration of Helsinki/Belmont Report, and informed written consent was obtained from adult participants and from guardians of children prior to study participation.

### Bacterial strains, plasmids, and media

*Salmonella enterica* serotype Typhi strain CT18 [22] was obtained from the *Salmonella* Genetic Stock Centre (Calgary, Alberta, Canada). Genomic DNA from this strain was used to construct a genomic inducible expression library in host strain *Escherichia coli* strain BL21(DE3). Bacterial strains were grown in Luria-Bertani (LB) media (with 50 µg/ml kanamycin for clones containing pET30 constructs) and maintained at −80°C in LB broth containing 15% glycerol.

### Patient and control sera

Individuals undergoing elective cholecystectomy in Kathmandu, Nepal were enrolled. At the time of cholecystectomy, a venous blood sample was stored and a bile sample was taken for microbiologic analysis as previously described [6]. Patients were categorized as (1) *S.* Typhi carriers if their bile culture was positive for *S.* Typhi; (2) *S.* Paratyphi A carriers if their bile culture was positive for *S.* Paratyphi A, or (3) cholecystectomy controls if their bile cultures were negative for any organism. Sera samples were also obtained from the following groups: (1) healthy Bangladeshi residents of Dhaka (a typhoid endemic area) enrolled at the International Centre for Diarrhoeal Disease Research, Bangladesh (icddr,b); and (2) acute (day 0–3) and convalescent sera (day 14–28) of Bangladeshi patients who presented to icddr,b with *S.* Typhi bacteremia [23]–[25].

### Construction of genomic inducible expression library

Genomic DNA was purified from *S.* Typhi strain CT18 using a Genomic DNA Isolation kit (Qiagen, Valencia, Ca), sheared using a Covaris sonicatior (Woburn, Ma) optimized to generate 0.5–1.5 kb DNA fragments, and resulting fragments were gel purified using the Qiagen Qiaquick Gel Extraction kit. After terminal overhangs were removed using End-It DNA end-repair kit (Epicenter Biotechnologies, Madison, WI), the blunt-end products were ligated into pET-30c vectors (Novagen, San Diego, CA) that had been digested with *Eco*RV and treated with calf intestinal alkaline phosphatase. The library was electroporated into *E. coli* DH5α and bacteria were plated onto selective LB media containing kanamycin. After overnight incubation at 37°C, the plates were scraped and the plasmid DNA from collected colonies was recovered using Qiagen Miniprep kit. *Eco*RI and *Kpn*I digestion was performed on a random sample of plasmids, and an insertion frequency greater than 80% and insert size between 500 to 1500 bp was verified. The plasmid DNA mixture was electroporated into *E. coli* BL21 (DE3), and collected colonies were stored in LB broth containing 15% glycerol.

### Screening for antigens uniquely expressed *in vivo* in *S.* Typhi carriers

Convalescent sera of 5 patients with bile cultures positive for *S.* Typhi were pooled, and adsorbed with *in vitro* grown *S.* Typhi strain CT18 and *E. coli* BL21 (DE3) [19]. Immunoblot techniques were used as previously described [19]. Briefly, the genomic library was plated on LB plates containing kanamycin to obtain a colony density of approximately 500 to 1000 clones per plate. After overnight incubation at 37°C, the resultant colonies were lifted off the plate using nitrocellulose membranes, and then the membranes were placed on LB media containing kanamycin and 1 mM isopropyl-β-D-thiogalactopyranoside for 4 hours at 37°C to induce transcription of insert DNA. Membranes were exposed to chloroform-soaked blotting paper to lyse bacteria, blocked for 1 hr using 5% milk in PBS with 0.25% Tween-20 (PBS/Tween), washed five times in PBS/Tween, and then incubated overnight with adsorbed sera at 1∶10,000 dilution. After membranes were washed 3 times with PBS/Tween, immunoreactive clones were detected using anti-human IgG conjugated to horseradish peroxidase (MP Biomedicals/Cappel, Aurora, OH) at a 1∶20,000 dilution, and immunoblots were developed with an enhanced chemiluminescence (ECL) kit (Amersham, Piscataway, NJ). Reactive clones were recovered from the master plates and saved as frozen glycerol stocks.

To confirm immunoreactive clones, secondary screening was performed comparing IgG immunoreactivity of the clones against *E. coli* BL21DE3 with an empty pET30c vector. Inserts of confirmed clones were sequenced to identify gene insert. Constructs designed to express the full length native protein were generated by amplifying the entire ORF of identified genes by PCR, and cloning these amplicons into pET30c as *Nde*I and *Not*I inserts. Immunoreactivity of these full ORF clones was compared to *E. coli* BL21DE3 with an empty pET30c vector. To assess immunoreactivity of identified antigens among the pertinent general population, immunoreactive clones were also screened using pooled sera of individuals living in a typhoid endemic area (Bangladesh). These sera were pre-adsorbed against *in vitro* grown *E. coli* BL21DE3, as described above, to reduce background reactivity against the host strain.

Functional classifications of identified proteins were assigned using published articles and available protein information resources, including J. Craig Venter Institute annotations (http://cmr.jcvi.org/tigr-scripts/CMR/CmrHomePage.cgi) and Pfam 26.0 (http://pfam.sanger.ac.uk/).

### Purification of YncE

YncE (STY1479) was PCR-amplified from *S.* Typhi strain CT18 and the product was cloned into Gateway vector pDONR221 using BP reaction kit according to manufacturer's instructions (Invitrogen). The full length sequence was verified and transferred from pDONR221 into the Gateway expression vector pDEST17 using LR reaction kit (Invitrogen) generating pDEST17His_6_-yncE. The reaction product was transformed first into *E. coli* DH5α, and then the recovered plasmid was transformed into the expression strain BL21AI. To overproduce His_6_-YncE, *E. coli* BL21AI (pDEST17His_6_-yncE) was grown in 250 mL LB broth containing ampicillin at 37°C until OD_600_ 0.6, and then expression of *his_6_-yncE* was induced by the addition of L(+) arabinose (0.2%). After 4 hours, the pellet was harvested by centrifugation, and the cells were lysed by sonication after resuspension in 15 mL lysis buffer (50 mM Tris Hcl, 5% glycerol, 0.1 M NaCl pH 8) containing 100 ug/ml lysozyme. Following centrifugation, the pellet was washed in lysis buffer with and without 1% Triton X-100, and the pellet was resuspended in 10 mL of 8 M urea, 50 mM NaH_2_PO_4_ and 300 mM NaCl (pH 7.4). His_6_-YncE was purified by HisPur Cobalt Resin (ThermoScientific, Rockford, Il) under denaturing conditions per the manufacturer's instructions. His_6_-YncE was then refolded by dialysis into 25 mM Tris-HCL 0.15 M NaCl, pH 8.0 using decreasing concentrations of urea. Product purity was assessed by polyacrylamide gel electrophoresis and Coomassie staining, and product identity was assessed by Mass spectrometry analysis. Protein concentration was determined via Coomassie (Bradford) Protein Assay Kit (ThermoScientific, Rockford, Il).

### Evaluation of serum IgG and IgA responses to YncE and *S.* Typhi capsular Vi antigen

To further characterize immunoreactivity of the antigen with the most prominent immunoreactivity in our initial screening, anti-YncE (STY1479) IgG and IgA responses were measured in the sera of 10 *S.* Typhi carriers, 3 *S.* Paratyphi A carriers, 8 patients at acute (day 0–3) and convalescent phase (day 14–28) of typhoid fever with confirmed *S.* Typhi bacteremia, 8 Nepalese controls undergoing elective cholecystectomy with negative bile cultures, and 8 healthy Bangladeshis, in duplicate. Plates were coated with 100 ng/well of YncE and then sera were added at a 1∶200 dilution. Bound antibody was detected with anti-human IgG or IgA conjugated with horseradish peroxidase (Jackson Laboratories, Bar Harbor, ME) at a 1∶1000 dilution, and peroxidase activity was measured with the substrate 2,2-azinobis (ethylbenzthiazolinesulfonic acid). To compare across plates, kinetic readings (mAb/sec) of samples were averaged, divided by kinetic readings (mAb/sec) of an in-house pooled standard (pooled sera of five *S.* Typhi carriers confirmed by biliary culture), and then multiplied by 100. Results were expressed as units (U). The Mann-Whitney U test was used to compare differences between groups.

For evaluation of anti-Vi IgG and IgA responses, ELISA plates were coated with 200 ng/well of Vi antigen (Sanofi Pasteur, Lyon, France). The above sera were applied at a 1∶100 dilution, and bound antibody was detected with anti-human IgG and IgA conjugated with horseradish peroxidase at a 1∶1000 dilution. Peroxidase activity was measured with the substrate 2,2-azinobis (ethylbenzthiazolinesulfonic acid). To compare anti-Vi responses across plates, duplicate kinetic readings of samples were averaged, divided by average kinetic readings of an in-house pooled standard, and then multiplied by 100, as described above. Results were expressed as units (U). Differences between groups were assessed using the Mann-Whitney U test.

## Results

### *S.* Typhi antigens identified by IVIAT

In the primary screen of over 120,000 clones, 565 clones were identified as immunogenic; 210 were confirmed by secondary screening. Sequence analysis of these inserts (many of which carried multiple potentially expressible ORFs) revealed 268 genes of interest with over 20% of genes identified multiple times, supporting validity of their identification and saturation of library screening. We subsequently sub-cloned the full coding sequences of 235 genes into individual expression plasmids, and identified 56 proteins with prominent IgG immunoreactivity using *S.* Typhi carrier sera, comparing immunoreactivity of expression clones to a clone containing an empty vector (Supplementary Table S1). Forty-eight of the identified genes are encoded on the chromosome of *S.* Typhi, 5 are encoded on the drug resistance plasmid pHCM1, and 3 on cryptic plasmid pHCM2. The most highly represented functional groups included proteins of unknown function and those involved in transport and binding, synthesis or salvage of ribonucleotides, and energy metabolism.

To assess the degree of immunoreactivity of antigens identified by IVIAT within the pertinent endemic-zone population, we screened the 56 immunoreactive clones against pooled sera of individuals living in a *S.* Typhi endemic area (Bangladeshi residents of Dhaka) [26]. Of these 56 proteins, 13 proteins had more prominent immunoreactivity when screened with sera of *S.* Typhi carriers compared to sera of healthy Bangladeshis. These 13 proteins included a number of putative membrane proteins, lipoproteins, and hemolysin-related proteins (Table 1). YncE, a possible ATP- binding protein, had the overall highest differential immunoreactivity compared to healthy endemic-zone control sera in our immunoblot assay.

**Table 1:** Proteins identified by IVIAT with higher IgG immunoreactivity in *S.* Typhi carriers compared to healthy typhoid-endemic zone controls.

Locus	Gene	Function
STY0712	*corC*	Hemolysin-related protein
STY1364		Hypothetical periplasmic protein
STY1479	*yncE*	Possible ATP-binding protein
STY2155	*sirA*	Invasion response-regulator
STY2248	*pduG*	PduG protein
STY2386		Putative lipoprotein
STY2454	*yejE*	Putative binding-protein-dependent transporter
STY2657	*xapB*	Xanthosine permease
STY3709	*purH*	Phosphoribosylaminoimidazolecarboxamideformyltransferase and IMP cyclohydrolase (bifunctional enzyme)
HCM1.137	*repE*	Replication initiation protein
HCM1.213c		Putative transposase
HCM2.0043		Hypothetical protein
HCM2.0069c		Hypothetical protein

### Anti-YncE serum responses as a diagnostic biomarker for asymptomatic S. Typhi carriage

**Figure 1:**
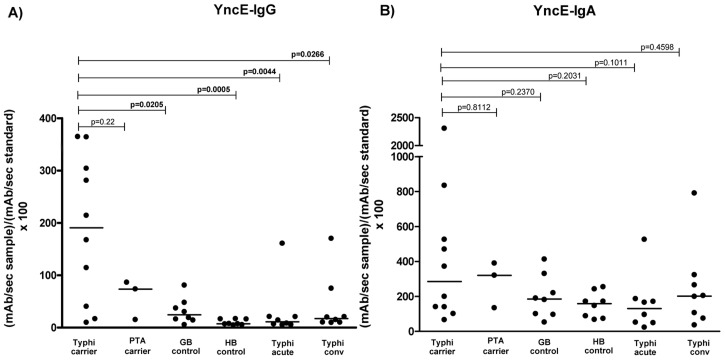
Characterization of anti- YncE immune responses. Anti-YncE IgG (A) and IgA (B) responses in *S.* Typhi carriers (Typhi carrier), *S.* Paratyphi A carriers (PTA carrier), Nepalese controls undergoing elective cholecystectomy with negative bile cultures (GB control), Healthy Bangladeshi controls (HB control), and patients at the day 0–3 acute (Typhi acute) and day 14–28 convalescent phase (Typhi conv) of typhoid fever with confirmed *S.* Typhi bacteremia.

To further characterize whether the immunoreactivity to YncE in *S.* Typhi carriers was specific, we also evaluated the immunoreactivity to YncE using sera of 5 groups of individuals: (1) *S.* Typhi carriers, (2) patients at the acute and convalescent phase of typhoid fever, (3) *S.* Paratyphi A carriers, (4) individuals who underwent cholecystectomy in Nepal whose bile cultures were negative for any pathogen, and (5) healthy controls from a typhoid endemic area (Dhaka, Bangladesh). We found significantly higher IgG immunoreactivity to YncE in *S.* Typhi carriers compared to bile culture-negative patients (p = 0.0205), healthy Bangladeshis (p = 0.0005), and patients at the acute and convalescent phases of typhoid infection (p = 0.0044 and p = 0.0266, respectively); there was a trend toward statistical significance when compared to *S.* Paratyphi A carriers (p = 0.21) (Figure 1A). Of the 10 *S.* Typhi carriers, 7 (70%) had an anti-YncE IgG response (Units >100). None of 8 bile culture negative controls (0%), 0 of 8 healthy Bangladeshis (0%), 0 of 3 *S.* Paratyphi A carriers (0%) and 1 of 8 (12.5%) Bangladeshis at the acute and convalescent phase of *S.* Typhi had an anti-YncE IgG response. Thus, in our small subset of patients, using a cut-off value of >100 Units (U), anti-YncE IgG had a sensitivity of 70%, and specificity of 100% when using endemic zone healthy individuals and cholecystectomy patients without detectable *S.* Typhi as controls. The specificity decreased to 95% if we included patients with acute typhoid fever.

Although, the values did not reach statistical significance, *S.* Typhi carriers also had a higher IgA immunoreactivity to YncE compared to our two control groups: bile culture-negative patients (p = 0.2370) and healthy Bangladeshis (p = 0.2031) (Figure 1B). There was no significant difference between the IgA immunoreactivity to YncE in S. Typhi carriers in comparison to patients convalescing from acute typhoid infection or *S.* Paratyphi A carriers.

### Comparison with anti-Vi serum responses

**Figure 2:**
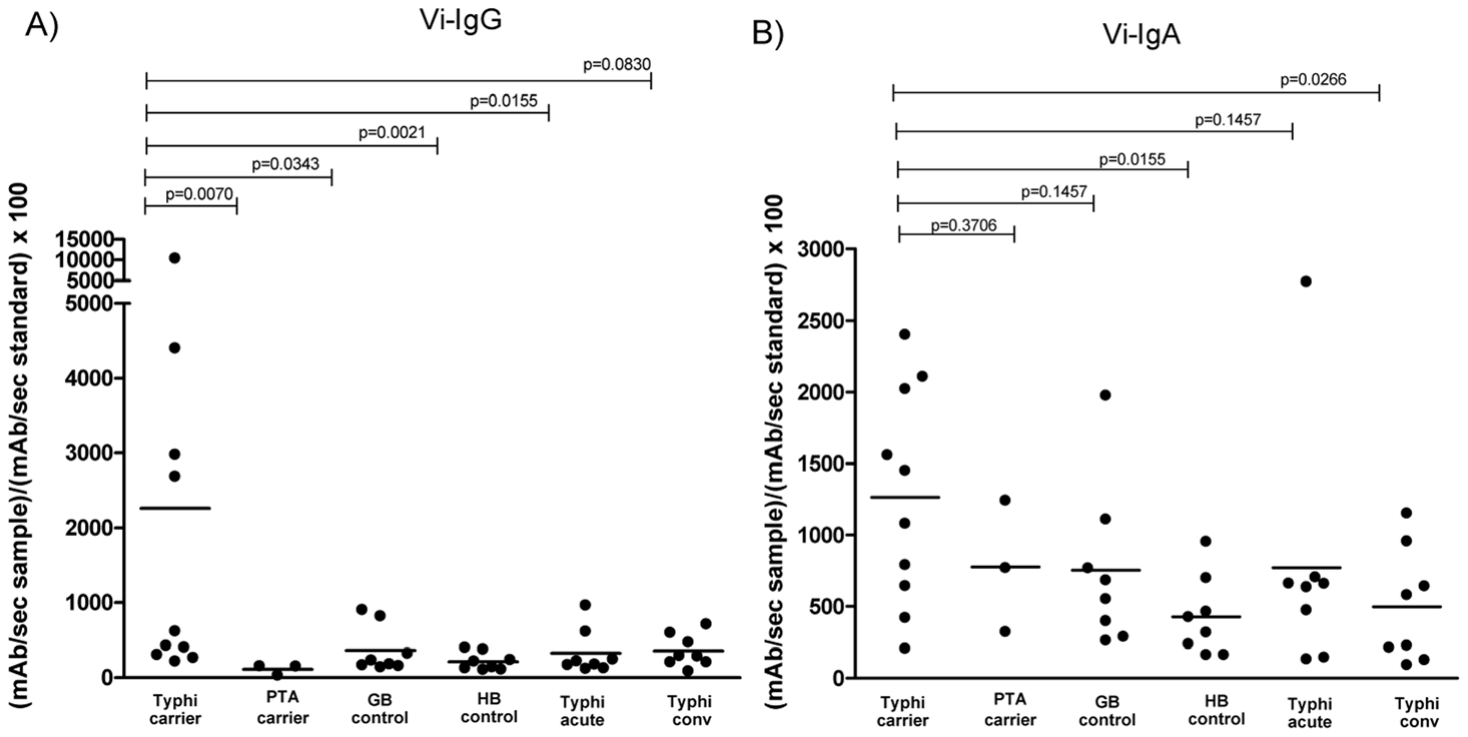
Characterization of anti- Vi immune responses. Anti- Vi antigen IgG (A) and IgA (B) responses were evaluated in *S.* Typhi carriers (Typhi carrier), *S.* Paratyphi A carriers (PTA carrier), Nepalese controls undergoing elective cholecystectomy with negative bile cultures (GB control), healthy Bangladeshi controls (HB control), and patients at the day 0–3 acute (Typhi acute) and day 14–28 convalescent phase (Typhi conv) of typhoid fever with confirmed *S.* Typhi bacteremia.

Since immune responses to *S.* Typhi Vi antigen have been the best characterized diagnostic method for identifying *S.* Typhi carriers to date, we also assessed the anti-Vi IgG and IgA responses in the same cohort of patients. We found significantly higher IgG immunoreactivity to Vi antigen in *S.* Typhi carriers compared to PTA carriers (p = 0.0070), bile culture negative controls (p = 0.0343), healthy Bangladeshis (p = 0.0021), and patients at the acute phase of typhoid infection (p = 0.0155). (Figure 2A). There was a trend toward statistical significance when the immunoreactivity of *S.* Typhi carriers to Vi antigen was compared to patients at the convalescent phase of typhoid infection (p = 0.0830) (Figure 2A). In our evaluation of IgA anti-Vi responses, we did find a significant difference in the immunoreactivity of *S.* Typhi carriers compared to healthy Bangladeshis (p = 0.0155), and patients convalescing from acute typhoid infection (p = 0.0266) (Figure 2B). There was no significant difference in immune responses between *S.* Typhi carriers and bile-culture negative patients, *S.* Paratyphi A carriers, or patients at the acute phase of typhoid infection. The sensitivity for anti-Vi IgG and IgA was 40% (cutoff value >1250 U) and 50% (cutoff value >1250 U), respectively. The specificity was 100% for IgG irrespective of controls. For IgA, the specificity was 97% when using endemic zone healthy individuals and cholecystectomy patients without detectable *S.* Typhi as controls. The specificity was 94% if patients with acute typhoid fever were included in the analysis.

### Combined YncE and Vi antibody measurements for the detection of S. Typhi carriers

In our analysis, using a cut-off value of >100 U of anti-YncE IgG and/or >1250 U anti-Vi IgA, we could identify 8 out of 10 *S.* Typhi carriers. There was no added benefit seen when pairing anti-YncE responses with anti-Vi IgG.

## Discussion

In our immunoscreen using IVIAT, we were able to identify 56 immunogenic *S.* Typhi proteins using the sera of *S.* Typhi carriers. Of these, 13 had higher immunoreactivity when screened with *S.* Typhi carrier sera compared to sera of endemic zone residents. These proteins represent a working list of candidate diagnostic biomarkers of asymptomatic *S.* Typhi carriage and their analysis may further our understanding of survival adaptations of *S.* Typhi in chronic carriers.

Human epidemiologic studies as well as murine models of *S.* Typhi carriage suggest that gallstones facilitate the development of the chronic carrier state [5]. In support of this, we identified SirA in our IVIAT screen, which is part of the two-component response regulator SirA-BarA [27]. In *S.* Typhimurium, this regulator plays a role in the down-regulation of genes involved in invasion (i.e. *Salmonella* Pathogenicity Island-1) when the bacterium is in the presence of bile [28], and mutations in *sirA* result in decreased biofilm formation on plastic surfaces [28]. The role SirA may play in human or murine *Salmonella* carriage, or why a cytoplasmic regulatory protein generated a humoral response, has yet to be characterized. Other proteins identified in the IVIAT screen may also affect carriage in the presence of gallstones.

Although *S.* Typhi may persist in the gallbladder in association with gallstones [3], *S.* Typhi likely has other niches of infection, including the gallbladder epithelium, biliary tree, and in macrophages of mesenteric lymph nodes [2], [3], [5], [16]–[18]. Proteins identified in our screen may play a role in persistence of *S.* Typhi within host cells or the stringent environment of bile. For instance, YejE is a putative permease that is thought to be a component of a putative ABC transporter system. YejE plays a role in survival within epithelial cells and in antimicrobial peptide resistance [29]. In both *S.* Typhi and *S.* Typhimurium, *yejE* expression is upregulated inside host macrophages [30], [31]. PduG is a protein encoded within the *pdu* operon that is part of the coenzyme B12-dependent 1,2-propranediol utilization pathway [32]. This operon is upregulated during acute *S.* Typhi and *S.* Paratyphi A infection in humans [24], [33], and may be associated with use of alternative carbon sources in the nutrient-limited environment of the *Salmonella*-containing vacuole within host cells [32]. We also identified PurH and XapB, which are proteins involved in purine biosynthesis and acquisition, respectively, by functional classification. In *S.* Typhimurium, PurH is associated with virulence [34], and we have previously shown that genes involved in purine synthesis are upregulated during acute typhoid infection in humans [24]. CorC is a hemolysin-related protein involved in magnesium and cobalt efflux, and is part of the CorA transporter system containing CorA-D [35]. CorA, with associated proteins, is required for efflux of Mg^2+^ [35]. CorA is required for *S.* Typhimurium virulence [36], and *corA* is expressed by *S.* Typhi during acute human infection [24]. However, while some information is known regarding the above mentioned *Salmonella* carrier-specific antigens, their potential role in carriage is presently unclear.

The majority of the genes identified by IVIAT encode for proteins with putative or unknown function. For example, STY2386 is an uncharacterized lipoprotein found uniquely in *Salmonella*. STY1364 is a hypothetical periplasmic protein in *S.* Typhi and *S.* Paratyphi A, and is rarely found in other *Salmonella* spp. STY1364 belongs to the structural classification of bacterial enterotoxins and is a subtilase cytotoxin subunit B-like protein. We previously identified STY1364 in *S.* Typhi infected patients using a separate immunoscreening technology (immunoaffinity proteomic-based technology, IPT) [23].

In our screening, YncE (STY1479) was the most immunoreactive antigen identified, and we thus focused our more detailed analysis of immunoreactivity on this antigen. YncE has a putative N-terminal signal sequence suggestive of export, with ATP and DNA-binding domains. y*ncE* is present in a number of *Salmonella* spp., and has orthologs in a number of other Gram-negative enteric organisms, including *Escherichia coli*, *Citrobacter* spp, and *Shigella* spp. In *E. coli*, YncE is secreted into the periplasm via the Sec-dependent pathway [37], and its expression is induced under iron-restricted conditions when repression by the Fur protein is relieved [38]. Its role in the pathogenesis of *Salmonella* infection has yet to be characterized. However, our results suggest that it may be involved in long-term persistence of the bacterium in chronic carriers.

In our analysis, we show that *S.* Typhi carriers have an IgG response to YncE that is not present in bile culture-negative controls in Nepal or healthy controls in Bangladesh. Although we did not reach statistical significance in this small pilot study, a similar trend was seen for IgA as well. One patient convalescing from acute typhoid infection had a detectable IgG anti-YncE response, and another had an IgA response. This may suggest that anti-YncE responses occur during acute disease; however, it should be noted that we do not know the current or future carrier status of the acute typhoid patients, and an elevated level of YncE during an episode of typhoid fever may represent an acute on chronic infection, or may be a marker of future progression to the chronic carrier state.

All of the identified genes except three (*xapB* and the two genes encoded on the cryptic plasmid pHCM2) are present in the genome of *S.* Paratyphi A sequenced strains ATCC 9150 and AKU 12601 based on <60% nucleotide identity. It is interesting then, that we did not see an IgG or IgA immune response to YncE in *S.* Paratyphi A carriers. This finding may suggest that *S.* Typhi and *S.* Paratyphi A use different strategies to persist in chronic carriers, that expression of YncE may be distinct in these two organisms, or that our study did not have sufficient power to examine this, as it included only three *S.* Paratyphi A carriers.

Despite this, in our small cohort of patients, measurement of anti-YncE IgG responses did appear to be both relatively sensitive and specific for identifying asymptomatic chronic *S.* Typhi carriers. Further studies will be needed to evaluate the diagnostic capabilities of anti-YncE responses in a larger and different cohort of patients. Of note, if such studies demonstrate higher anti-YncE IgA levels in *S.* Typhi carriers than in control groups, that information could support consideration of a salivary diagnostic to facilitate community-based screening for carriage.

The other antigens identified in our IVIAT analysis may also be useful diagnostic biomarkers of *S.* Typhi carriage, and the sensitivity of carrier detection may be improved when responses against these or anti-Vi responses are paired with responses to YncE. For example, in our analysis, using a cut-off value of >100 U of anti YncE IgG and/or >1250 U anti-Vi IgA, we could identify 8 out 10 *S.* Typhi carriers. There was no added benefit seen when pairing anti-YncE responses with anti-Vi IgG. Another potential pairing could include a marker of biliary tract inflammation such as elevated bilirubin values, since *S.* Typhi carriage is often associated with chronic inflammation of the gallbladder [5]. We did not assess this parameter in this study.

Our study has a number of limitations. First, the number of patients involved in our study is small, although it should be noted that our analysis is the largest study involving immunoproteomic screening and pilot confirmation of the carriage state that includes appropriate control groups. A second limitation is that IVIAT identifies proteins that are uniquely expressed *in vivo* compared to standard *in vitro* culturing, and that also induce an antibody response. Proteins that induce cellular responses and/or that are expressed both *in vivo* and *in vitro* may also play a role in the pathogenesis of chronic carriage and serve as useful biomarkers for asymptomatic carriage. In addition, altering *in vitro* culturing conditions may also change the expression profile of *S.* Typhi, thereby changing the comparison groups. In addition, IVIAT does not identify non-protein antigens that may also be useful in diagnostic assays. However, despite these limitations, we have used IVIAT to identify a subset of immunoreactive antigens in *S.* Typhi carriers, including YncE. Further evaluation of YncE and other identified antigens could lead to the development of improved diagnostic assays to detect asymptomatic *S.* Typhi carriers in typhoid endemic zones, and analysis of YncE, along with other identified antigens, could lead to an improved understanding of host-pathogen interactions during chronic carriage of *S.* Typhi in humans.

## Supporting Information

Table S1 Proteins identified by IVIAT with higher IgG immunoreactivity in *S.* Typhi carriers compared to immunoreactivity to empty vector.
(XLS)
